# Dynamic identification of important nodes in complex networks based on the KPDN–INCC method

**DOI:** 10.1038/s41598-024-56226-8

**Published:** 2024-03-09

**Authors:** Jieyong Zhang, Liang Zhao, Peng Sun, Wei Liang

**Affiliations:** 1 Information and Navigation College, Air Force Engineering University, Xi’an, 710077 China; 2 No. 96872 Troops of PLA, Baoji, 721000 China

**Keywords:** Complex networks, Dynamic attack, Node importance, INCC, KPDN, Complex networks, Nonlinear phenomena

## Abstract

This article focuses on the cascading failure problem and node importance evaluation method in complex networks. To address the issue of identifying important nodes in dynamic networks, the method used in static networks is introduced and the necessity of re-evaluating node status during node removal is proposed. Studies have found that the methods for identifying dynamic and static network nodes are two different directions, and most literature only uses dynamic methods to verify static methods. Therefore, it is necessary to find suitable node evaluation methods for dynamic networks. Based on this, this article proposes a method that integrates local and global correlation properties. In terms of global features, we introduce an improved k-shell method with fusion degree to improve the resolution of node ranking. In terms of local features, we introduce Solton factor and structure hole factor improved by INCC (improved network constraint coefficient), which effectively improves the algorithm’s ability to identify the relationship between adjacent nodes. Through comparison with existing methods, it is found that the KPDN–INCC method proposed in this paper complements the KPDN method and can accurately identify important nodes, thereby helping to quickly disintegrate the network. Finally, the effectiveness of the proposed method in identifying important nodes in a small-world network with a random parameter less than 0.4 was verified through artificial network experiments.

## Introduction

Currently, research on identifying important nodes in dynamic complex networks mainly focuses on two directions: first, using time series analysis to predict the dynamic changes in network topology based on edges^1–4^. For example, Yujing Shi et al.^5^ utilized time synchronous control to demonstrate the finite-time stability of dynamic network error systems, while Ting Zhang et al.^6^ also pointed out the challenges faced in link prediction, such as the difficulty of applying static network centrality metrics to dynamic networks. This line of research primarily focuses on the changing patterns of edges in dynamic networks. Second, the direct identification of important nodes that play crucial roles in dynamic networks. Given that link prediction has already been extensively studied, we intend to focus on conducting research on identifying important nodes in dynamic networks.

There has already been extensive research on identifying important nodes in static networks. Various centrality metrics like degree centrality, closeness centrality, and betweenness centrality as well as their fusion methods^7–11^ are more applicable to stable topological structures in real physical environments. However, the difficulty of recognizing critical nodes increases significantly when network structures change dynamically, especially in areas like network protocols and network security. To address this challenge, some attempts have been made by scholars. Fu’s team^12^ fused multiple centrality metrics to improve identification efficiency. Shao’s team^13^ used local neighborhood priority asynchronous H computation to accelerate convergence speed. Ruan’s team^14^ constructed a two-step neighborhood model to evaluate node importance, which can be applied to large-scale dynamic networks.

Of course, there are also studies on dynamic network analysis based on real-world scenarios. For instance, Zhang Hui’s team^15^ incorporates dynamic network representation learning into recommendation systems and proposes an algorithm that optimizes node heterogeneity in bipartite networks. Tan Shiyin et al.^16^ address the existing issues in link prediction research by studying link prediction algorithms in both dynamic networks and heterogeneous networks, constructing models that accurately predict the relationships between network edges. Zhao Danling et al.^17^ propose evaluation methods for the contribution rate of weapon equipment systems based on combat tasks and the effectiveness contribution rate of weapon equipment systems based on system simulation during combat processes. The transition between dynamic networks and static networks is also crucial. Wu Jiaming et al.^18^ propose three dynamic network representation methods based on the topological structure changes, large-scale networks, and overall characterization of networks using static snapshots. Srijan et al.^19^ present a more efficient real-time explicit embedding method for updating user-project interaction networks with dynamic evolution. Meanwhile, some scholars have proposed new centrality measures based on the positive and negative effect^20^ of the clustering coefficient in complex networks. They have also developed a deep semi-supervised detection method^21^ based on pointwise mutual information for community detection. In this chapter, we intend to follow this approach and propose a method that combines multiple indicators to identify nodes. At the same time, drawing inspiration from the time-slice snapshot concept, we design an identification algorithm that uses indicator fusion in multiple static time slices to handle network dynamics. For further advancements, additional references can be found in^22^.

There are several issues with existing methods:

1. There is a greater emphasis on the identification of important nodes in static networks compared to dynamic networks. Many studies do not specifically discuss the use of dynamic methods and only consider them as a supplement to static methods, lacking targeted exploration of dynamic approaches.
2. Some methods exhibit good global and local characteristics during static network analysis. However, as network nodes are continuously removed, pre-analyzing the remaining network may not yield equally effective results.
3. Certain algorithms perform well in random-like networks, such as small-world networks, but they may not provide better resolution for networks with lower randomness.

Therefore, based on the basic analysis of the aforementioned issues, the main contributions of this paper are as follows:

1. In order to fully understand the stable operation of complex systems and further explore the failure propagation process and underlying mechanisms in real-world complex systems, we propose a node importance evaluation model that considers the dynamic characteristics of networks. Based on this model, we conduct cascade failure simulations under different attack strategies, using three metrics—maximum connected component, maximum remaining edges, and subnet sensitivity—to quantify the resilience of complex networks against cascade failures.
2. In this proposed model, we also design a method called KPDN–INCC, which is based on the evaluation of important nodes in dynamic complex networks. We demonstrate the results of cascade failure simulations with various node removal strategies using multiple real-world networks. From the experimental analysis of real network data, it can be observed that the proposed method exhibits good performance in the cascade failure results after node removal. Particularly, it demonstrates advantages in identifying node importance and resolution, especially in small-world network experiments.
3. By improving the KPDN algorithm, the static algorithm can participate in the process of identifying the importance of dynamic nodes. This achieves complementarity between the KPDN algorithm in static node importance assessment and dynamic node importance evaluation.

The organization of the outline is as follows: “Materials and methods” section mainly introduces the algorithm we proposed, while also introducing several classic important node identification algorithms for comparison; “Evaluation indicators” section introduces two metrics we used to evaluate the effectiveness of dynamic node importance; “Network data” section introduces the several real-world networks and one artificial network used in our experiments; “Experimental analysis” section analyzes the simulation results on the real-world and artificial networks, while detailing comparing the results of the artificial network under different experimental conditions; “Conclusion” section summarizes.

## Materials and methods

**Figure 1:**
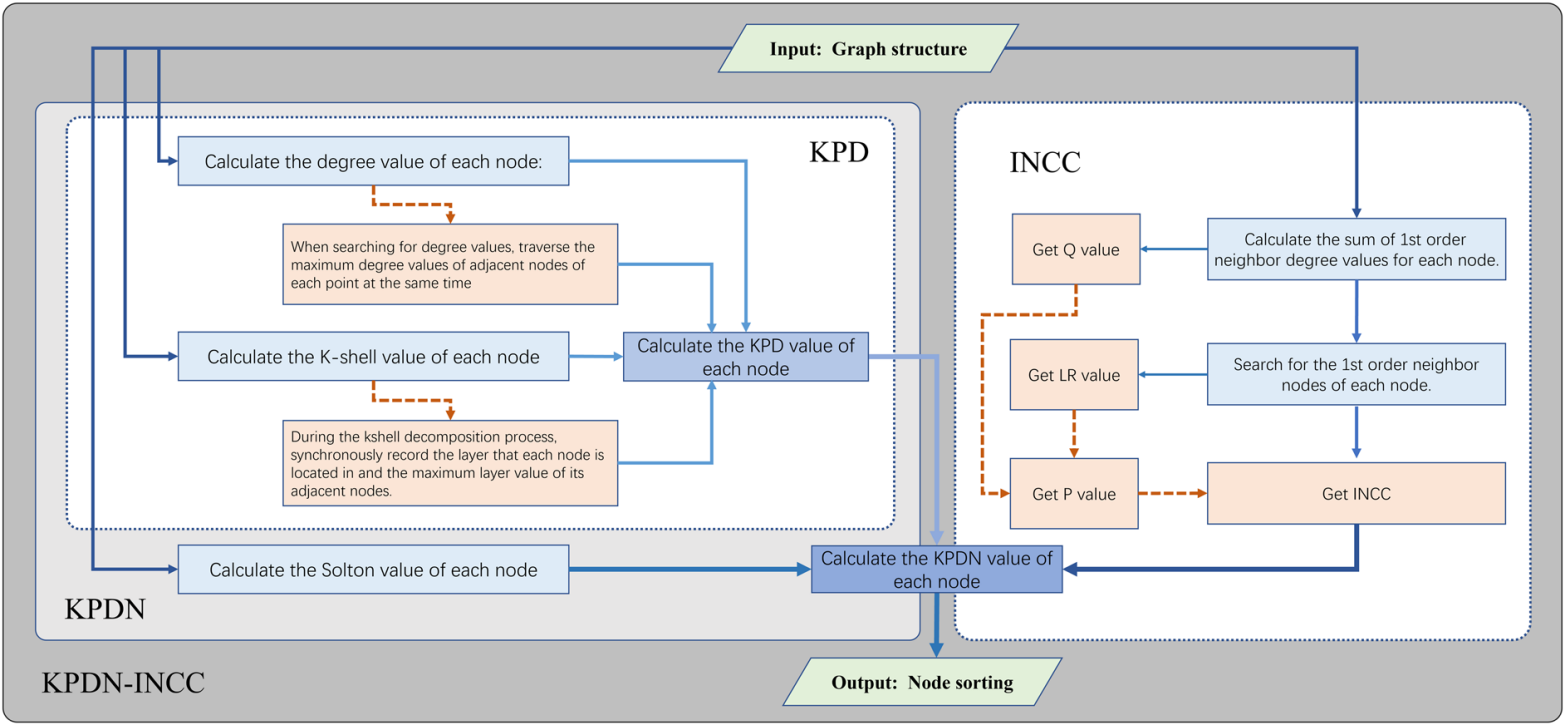
Flowchart of the KPDN–INCC method.

To address the problems with identifying important nodes in complex network dynamics, this paper draws inspiration from references^23^ and^24^ and proposes a KPDN–INCC method (A K-Shell methods positioned with Degrees, Neighbors and INCC). This method comprehensively considers global and local features by applying the K-shell method globally to stratify the network, and recording the separation order during each K-shell stratification process as the basis for secondary sorting. We then judge the correlation between each node and its neighboring nodes according to the Solton index, and conduct tertiary sorting. Finally, based on the changes in the remaining network after each node deletion operation, we introduce the INCC factor for quaternary sorting to distinguish the importance of dynamic nodes. Since the KPDN–INCC method does not contain free parameters and the neighbor information for each node can be recorded simultaneously when traversing its degree values, most calculations are based on operations between a node and its neighbors, and the improved k-shell method is also recorded along the way during one k-shell expansion process, thus having low computational complexity. Also, by introducing the INCC factor, the structural metrics of the network can be better preserved during the dynamic node deletion process, thus effectively judging the structural importance of nodes in the remaining network. More details refers to Fig. 1.

The specific steps are as follows:

*Step 1*: First, use the k-shell method to record which k-shell layer each node belongs to. Next, when nodes in the same k-shell layer are peeled off sequentially, record the order in which each node is peeled off as well as the layer number and sequence number. Finally, for the set of nodes in each k-shell layer with the same peel-off sequence layer, sort them according to the proportional degree value size of nodes. It can be expressed by the mathematical formula (1) as follows:

1 $$ KPD_{i} = ks_{i} + k_{i} + \frac{{k_{i} }}{{k_{N(i)\max } + 1}}*\frac{{l_{i} }}{{l_{\max } + 1}}*MAX\left( {1,ks_{i|next} - ks_{i} } \right) $$

where $ks_{i}$ is the $k - shell$ value of node $i$, $k_{i}$ is the degree value of node $i$, $k_{N(i)\max }$ is the maximum degree value of node $i$’s neighbor nodes, $l_{i}$ is the peel-off order of node $i$ under the iterative peel-off operation in the same $ks_{i}$ layer, $l_{\max }$ is the maximum peel-off sequence number under the same $ks_{i}$ layer; $ks_{i|next}$ is the maximum $k - shell$ value among the neighbors of node $i$. In particular, to avoid the maximum $ks_{i}$ layer not participating in the calculation, when a node is in the maximum $k - shell$ layer, the $ks_{i|next} - ks_{i}$ value is automatically set to 1.

*Step 2*: We calculate the relationships between the obtained neighboring nodes of each node. Here, we draw inspiration from previous research and define the edge strength between two connected nodes based on the local topological information of the network, such as the Solton index proposed in reference^25^, with the formula as follows:

2 $$ S_{ij} = \frac{{\left| {N\left( i \right) \cap N\left( j \right)} \right|}}{{\left| {N\left( i \right) \cup N\left( j \right)} \right|}}\;\;{\text{or}}\;\;S_{ij} = \frac{{\left| {N\left( i \right) \cap N\left( j \right)} \right|}}{{\sqrt {k_{i} *k_{j} } }} $$

*Step 3*: Inspired by reference^24^, we calculate the structural hole index of the entire network when nodes are dynamically deleted, and introduce the INCC (Improved Network Constraint Coefficient) factor to capture the indirect effects on the nearest neighbors and secondary neighbors of each node after the network changes due to dynamic node deletion. The formula is as follows:

3 $$ INCC_{i} = \sum\limits_{j \in \Gamma (i)} {\left[ {p^{\prime}_{ij} + \sum\limits_{k = 1,k \ne i,j}^{n} {p^{\prime}_{ik} p^{\prime}_{kj} } } \right]^{2} } $$

where $p^{\prime}_{ij} = \frac{{Q_{j} }}{{LR_{i} }}$, $Q_{j} = \sum\limits_{w \in \Gamma (j)} {N_{w} }$, $LR_{i} = \sum\limits_{j \in \Gamma (i)} {Q_{j} }$, $N_{w}$ is the sum of degree values of node $j$’s neighbor nodes, $\Gamma (j)$ represents the set of node $j$’s neighbor nodes, $\Gamma (i)$ represents the set of node $i$’s neighbor nodes.

**Figure 2:**
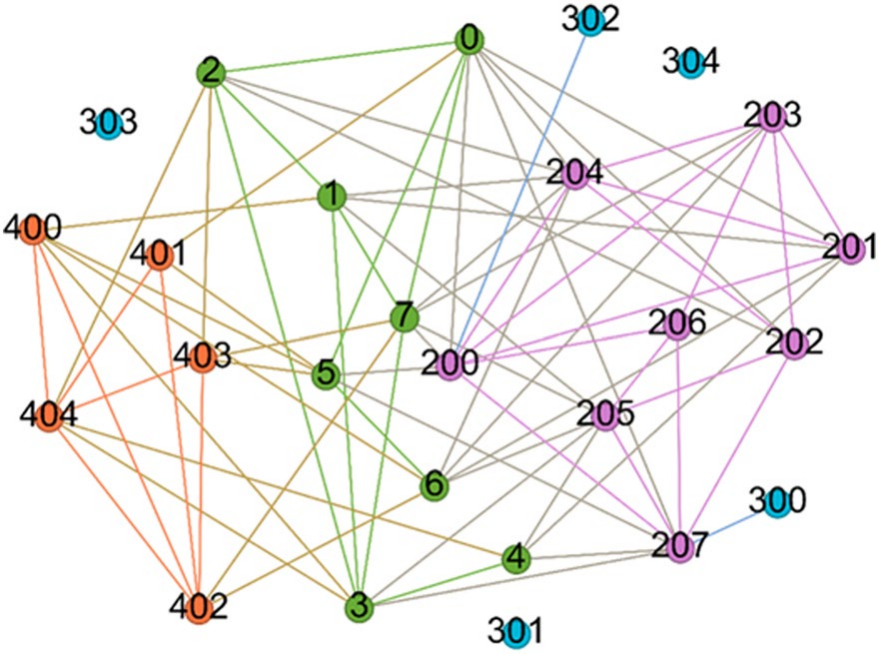
Calculation example of the KPDN–INCC method.

As shown in Fig. 2, a randomly generated demo case is selected. We choose the KPD method, KPDN method, Degree method, K-shell method and KPDN–INCC method to perform numerical calculations according to the formulas respectively. For more details, please refer to Attachment 1.

It can be seen from the figure that the effect of the KPDN–INCC method proposed in this chapter is quite obvious. After deleting 8 nodes, the effect may be average and there is basically no noticeable change. However, when 13 nodes are deleted, it is clear that the remaining network size obtained through the KPDN–INCC method is only 5.

**Figure 3:**
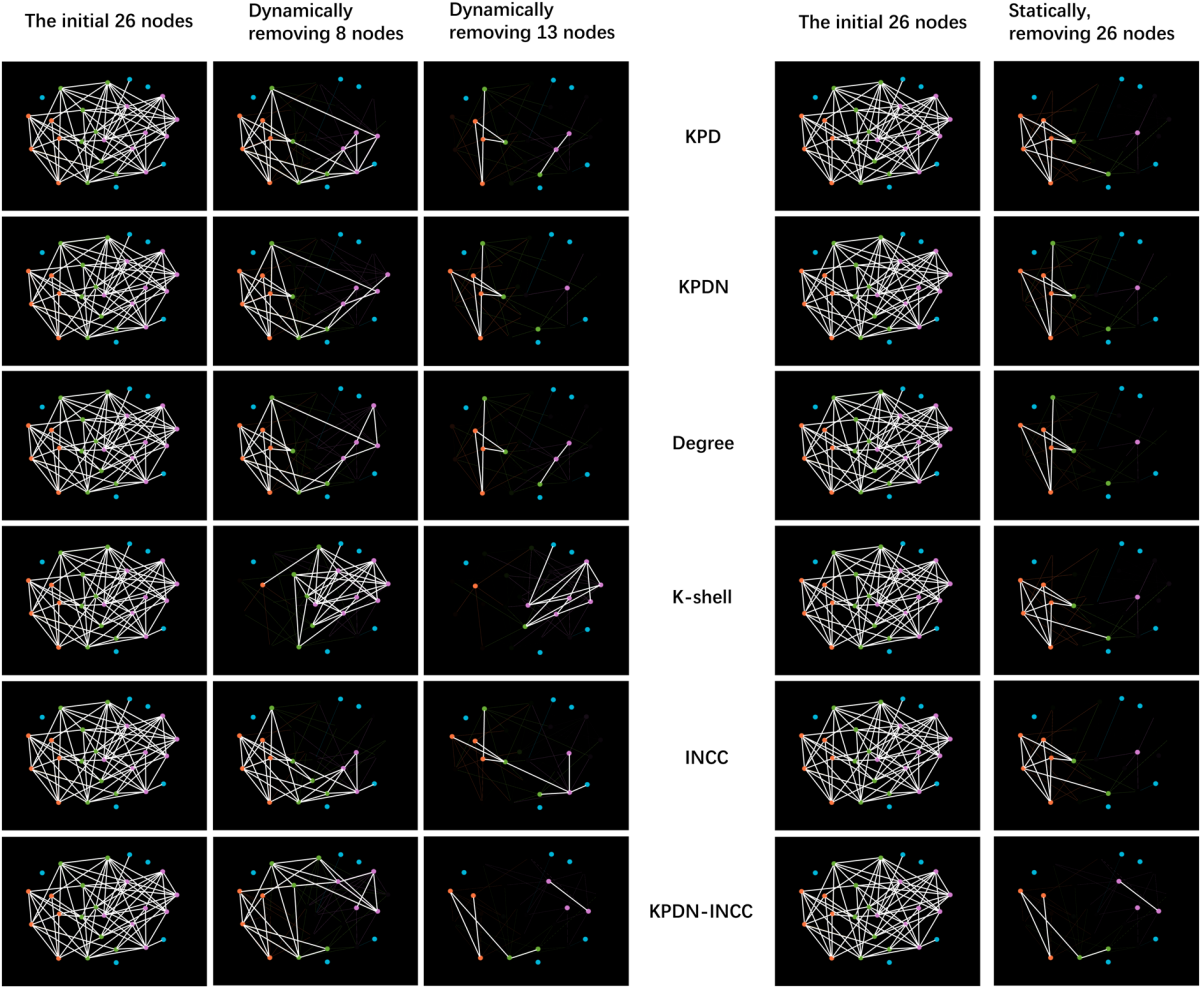
Comparison of static and dynamic important node identification results for six methods.

Dynamic important node identification methods generally perform better than static methods. However, some static methods can be equally as effective as dynamic ones. For this example, the KPDN and KPDN–INCC static and dynamic results are consistent. But some dynamic methods underperform static alternatives, like the K-shell and INCC methods. So dynamic techniques provide an alternative to static approaches. But whether to use dynamic or static identification requires case-by-case validation based on the actual network. In order to have a more intuitive understanding of the method proposed in this paper and other existing methods, we compare the method proposed in this paper with five classical methods to observe the results of dynamic important nodes. The image shows the remaining network after dynamically removing 8 nodes and 13 nodes respectively. The results are shown in Fig. 3.

Figure 2 depicts a randomly generated small-world network image, and the randomness is intended to ensure that the algorithm proposed in this paper exhibits a certain degree of universality within small-world networks. Figure 3, on the other hand, compares the effects of different algorithms for node removal, presenting the results of different node removal methods under both static and dynamic removal scenarios. To provide readers with a more intuitive understanding, we used the Gephi software to display the effects of node removal at different stages. As indicated in the third column from the left in Fig. 3, six different node removal strategies demonstrate distinct performances during the process of dynamic node removal. From the size of the remaining largest connected component, it can be observed that the KPD method yields a size of 5 after removing 13 nodes, while the KPDN method yields a size of 6, the DC method yields a size of 5, the K-shell method yields a size of 8, and the INCC method yields a size of 9. In contrast, the combined KPDN–INCC method proposed in this paper yields a size of 4. Similarly, using the maximum remaining edges as an indicator, the results are as follows: KPD: 7, KPDN: 7, DC: 7, K-shell: 11, INCC: 8, and the proposed KPDN–INCC method: 5. Therefore, it can be concluded that the method proposed in this paper demonstrates favorable performance in node importance assessment.

## Evaluation indicators

### Maximum connected subgraph

The maximum connected subgraph is another metric to evaluate the performance of ranking methods. It is defined as follows:

4 $$ P_{Subnet} = \frac{R}{N} $$

where $R$ is the size of the maximum connected subgraph after removing the node, and $N$ represents the size of the original connected network graph. Therefore, the smaller the $P_{Nodes}$ value, the smaller the ratio of the maximum connected subgraph to the original graph after removing the node, which proves the better effectiveness of removing the node.

### Average remaining edges

The average remaining edges is an important measurement indicator that allows us to better understand the remaining connectivity of the network when a node is destroyed. It can be used not only for subsequent path analysis, but also as an important influencing indicator of network repair costs. The calculation method is as follows:

5 $$ P_{Edges} = \frac{E}{M} $$

where $E$ is the total number of remaining edges in the network after removing a node each time, and $M$ is the initial number of connected edges in the network.

### Subnetwork sensitivity

The calculation for network sensitivity rate is as follows:

6 $$ S = \sum\limits_{s < \sigma } {\frac{{n_{s} s^{2} }}{n}} $$

where $n_{s}$ is the number of components of size $s$, $n$ is the number of nodes in the network, and $\sigma$ is the threshold value for judging the remaining fragments after the network is destroyed. As network nodes are gradually removed, the network is decomposed into many small disconnected parts, namely small-sized network fragments. When the network disintegrates to a certain extent, there is often a peak value of sensitivity $S$ on the image, and the appearance of peak value also means that the original network has been decomposed to the maximum extent into fragment groups less than or equal to the threshold $\sigma$.

## Network data

The following are seven datasets: Facebook_1912, Aves-wildbird-network, US-airports, Soc-hamsterster, Fb-pages-tvshow, P2p-Gnutella08 and Fb-pages-government, with detailed information as shown in Table 1.

**Table 1:** Specific parameters of real-world networks.

Dateset	Node	Edges	Average degree	Density (%)	Average clustering coefficient (%)
Facebook_1912	747	30,025	40.1941098	5.388	63.54
Aves-wildbird-network	1133	5451	4.8111209	0.43	22.02
US-airports	1574	17,215	10.9371029	0.695	50.42
Soc-hamsterster	2426	16,630	6.8549052	2.83	53.75
Fb-pages-tvshow	3892	17,262	4.4352518	0.114	37.37
P2p-Gnutella08	6301	20,777	3.2974131	0.05	1.087
Fb-pages-government	7057	89,455	12.6760663	1.796	41.09

These seven network datasets cover the domains of society, biology, technology and more, and can be used for complex network analysis and model research. At the same time, in order to facilitate the study of network characteristics that are more suitable for the method proposed in this chapter, we select small-world networks as our artificial network dataset.

## Experimental analysis

### Existing classical algorithms

In this paper, we compare the performance of the proposed method with several well-known classical algorithms. These algorithms include: K-shell, DC, CI, WL, HC, INCC, DWT, KPDN, Pagerank and Random methods. For more details on the comparative algorithms, please refer to Appendix 3.

### Analysis of real-world networks

In this section, we compare the performance of the proposed method with other methods in real-world networks. The dynamic analysis methods are highlighted as shown in the figure, which studies the robustness of networks in node removal.

**Figure 4:**
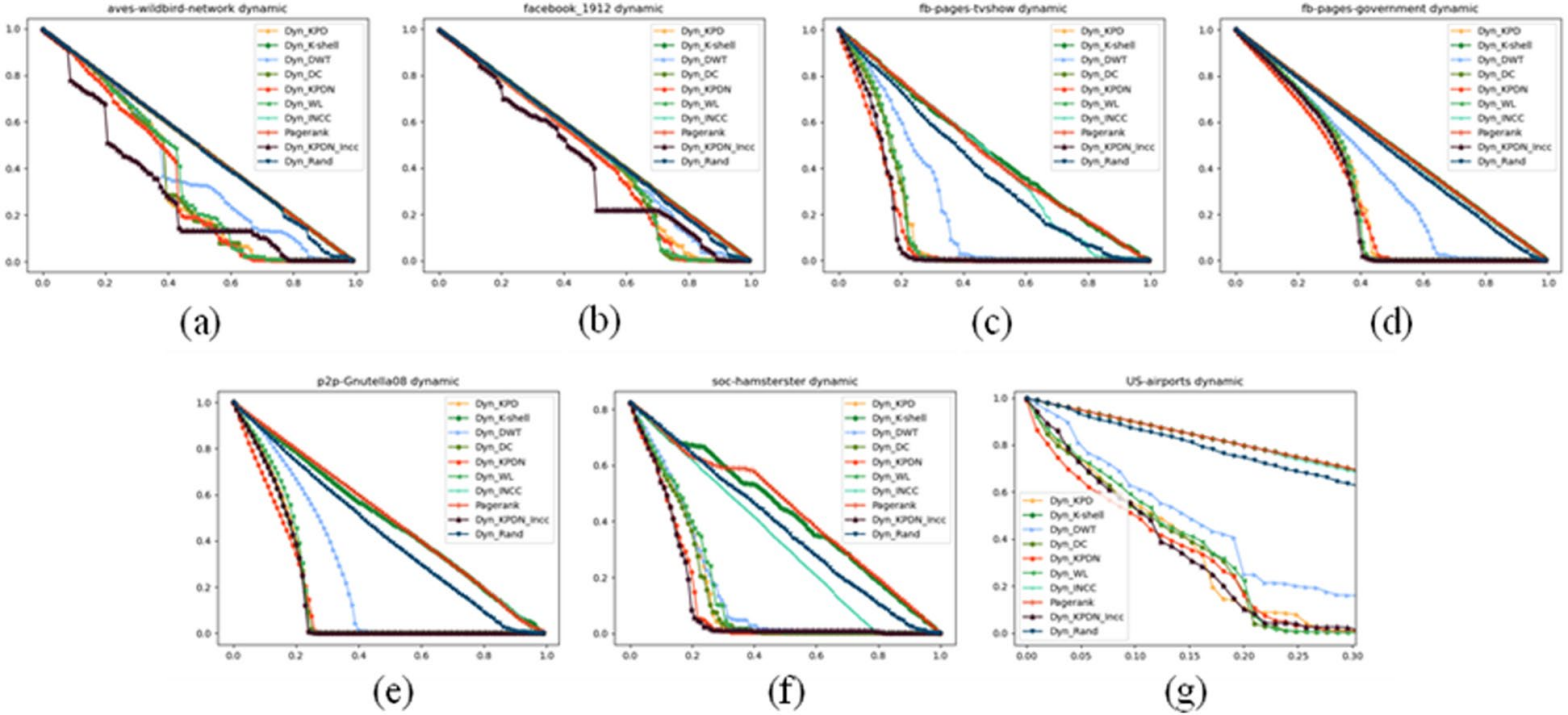
Comparison of subnetwork connectivity coefficients of real networks. Figures (**a**)–(**f**) are: (**a**) Facebook_1912 (**b**) Aves-wildbird-network (**c**) US-airports (**d**) Soc-hamsterster (**e**) Fb-pages-tvsho (f) P2p-Gnutella08 (g) Fb-pages-government.

**Figure 5:**
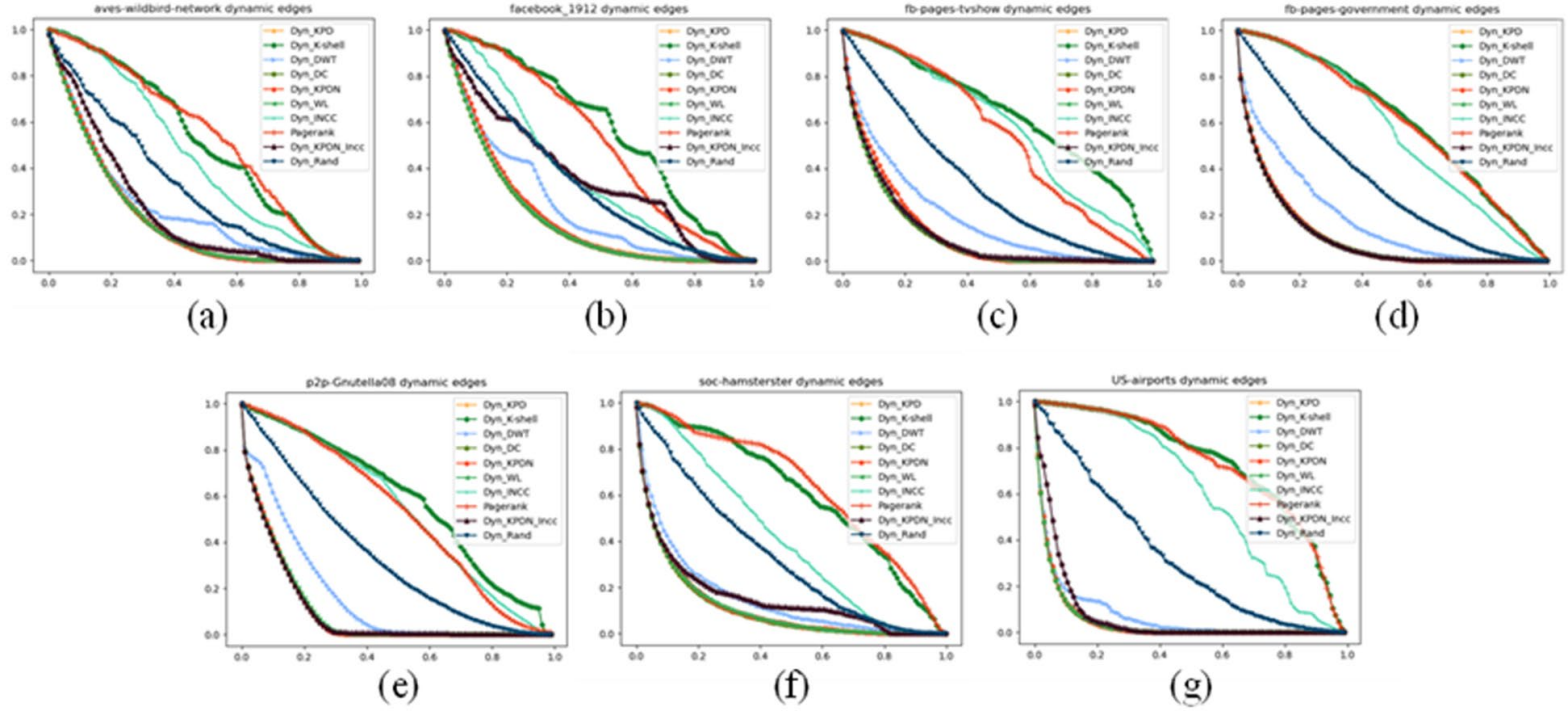
Comparison of remaining edge scales of subnetworks in real networks. Figures (**a**)–(**f**) are: (**a**) Facebook_1912 (**b**) Aves-wildbird-network (**c**) US-airports (**d**) Soc-hamsterster (**e**) Fb-pages-tvsho (**f**) P2p-Gnutella08 (**g**) Fb-pages-government.

**Figure 6:**
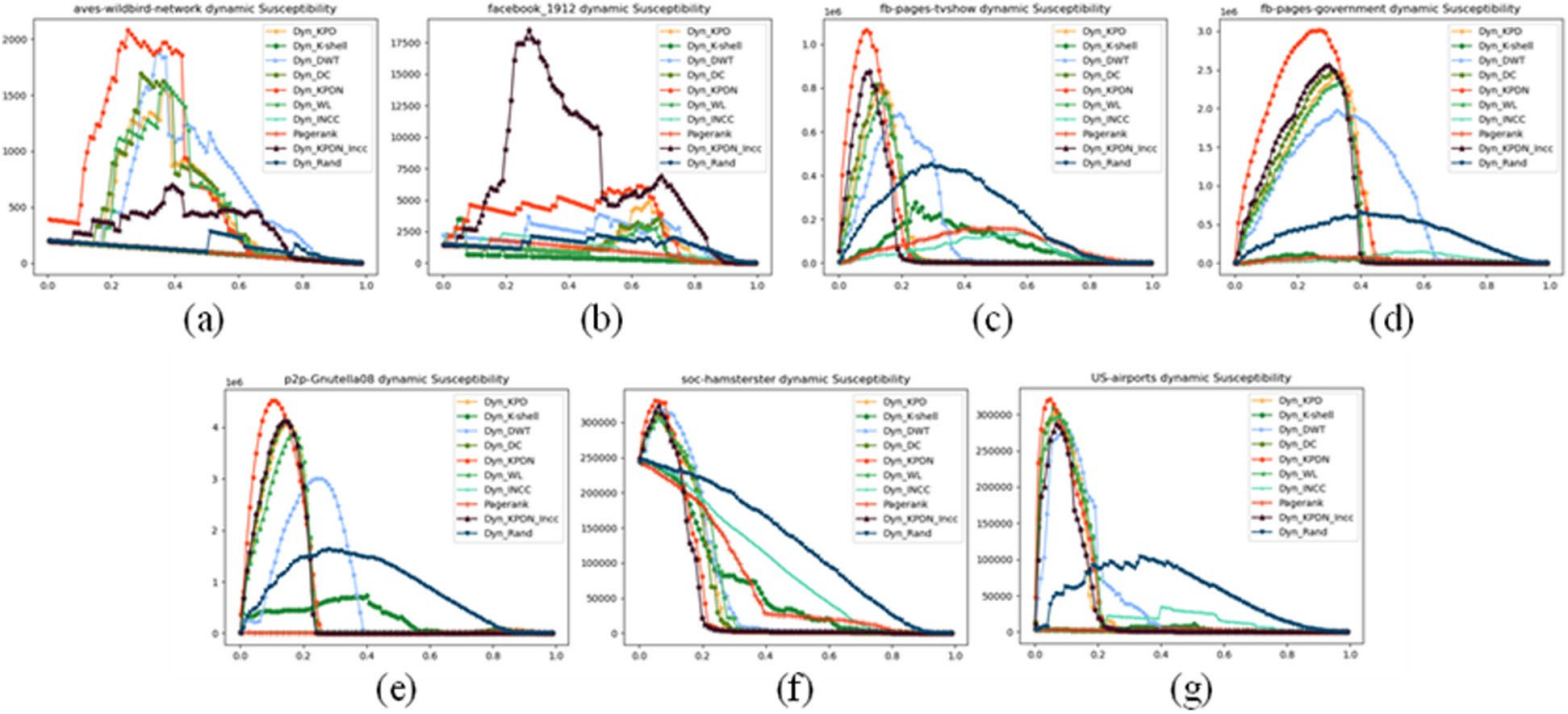
Comparison of subnetwork sensitivity of real networks. Figures (**a**)–(**f**) are: (**a**) Facebook_1912 (**b**) Aves-wildbird-network (**c**) US-airports (**d**) Soc-hamsterster (**e**) Fb-pages-tvsho (**f**) P2p-Gnutella08 (**g**) Fb-pages-government.

Figures 4, 5 and 6 depict the different experimental results obtained when using real networks as datasets under three different metrics. Taking Fig. 4 as an example, each subfigure (i.e., Fig. 4a–g) shows the proportion of removed nodes relative to the total number of nodes on the horizontal axis, and the proportion of the remaining largest connected subgraph to the initial network size on the vertical axis. In order to better distinguish the actual effects of different algorithms, the black line represents the KPDN–INCC algorithm proposed in this paper, while the red curve represents the original KPDN algorithm. From Fig. 4a, b, and f, it is evident that the algorithm proposed in this paper can rapidly reduce the size of the largest connected subgraph with a relatively small proportion of removed nodes. Similarly, from Fig. 4c, d, e, and g, it can be observed that although initially reducing the proportion of the largest connected subgraph to 50% may not be optimal, it is relatively fast compared to all algorithms. Furthermore, even when the subnet connectivity coefficient rapidly decreases to below 10%, the KPDN–INCC method still requires the smallest proportion of removed nodes (i.e., for images with vertical axis values less than 10%). In conclusion, this further validates the accuracy of the method proposed in this paper for dynamically assessing node importance. Similar analysis can be applied to Figs. 5 and 6. (In the experimental conditions proposed in this paper, based on the results of preliminary experiments, it was found that the effectiveness of node deletion in dynamic important node evaluation method is best when deleting nodes one by one. This is hereby noted.)

As shown in Fig. 4a,b, the dynamic node identification effect of the method proposed in this paper is very significant. Figure 4c–g provide some supplements to the KPDN method.

According to the actual data, it can be observed from Fig. 5a–g that the change of the subgraph edge remaining rate indicator of the method in this paper is quite obvious. Of course, this indicator works best under the dynamic degree attack strategy. Therefore, this indicator does not have an absolute advantage in general, but from the figure we can also see that even if it is not the optimal remaining edge attack strategy, it can maintain a relatively fast remaining edge strategy while quickly reducing the maximum connected subgraph of the network.

The threshold set in this paper is 100% of the original network size. When the sensitivity index peaks after removing a certain proportion of nodes, it means that each method has maximally decomposed the original network into fragments below the threshold size. Then as the fragments further shrink, the sensitivity enters a downward stage.

When comparing Figs. 5b and 6b, it can be seen that even if it is not the optimal remaining edge attack strategy, it can maintain a relatively fast remaining edge strategy while quickly reducing the maximum connected subgraph of the network. (Therefore, there is some uncorrelation between the several evaluation criteria selected in this paper.) Of course, the results of Fig. 6a, c–g may not be as significant as the KPDN method, which is why, as we mentioned at the beginning, the method proposed in this paper is intended as a supplement to the KPDN method.

### Analysis of artificial networks

**Figure 7:**
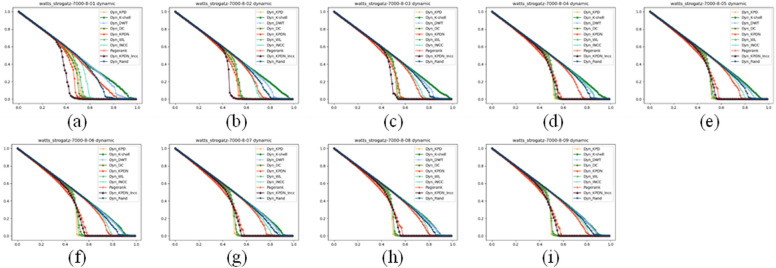
The maximum connected subgraph of small-world networks under different attack strategies.

**Figure 8:**
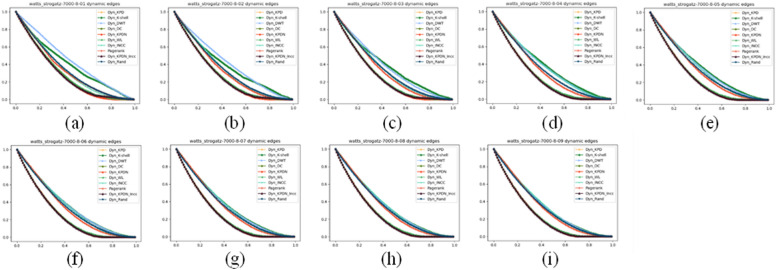
The maximum remaining edges of small-world networks under different attack strategies.

**Figure 9:**
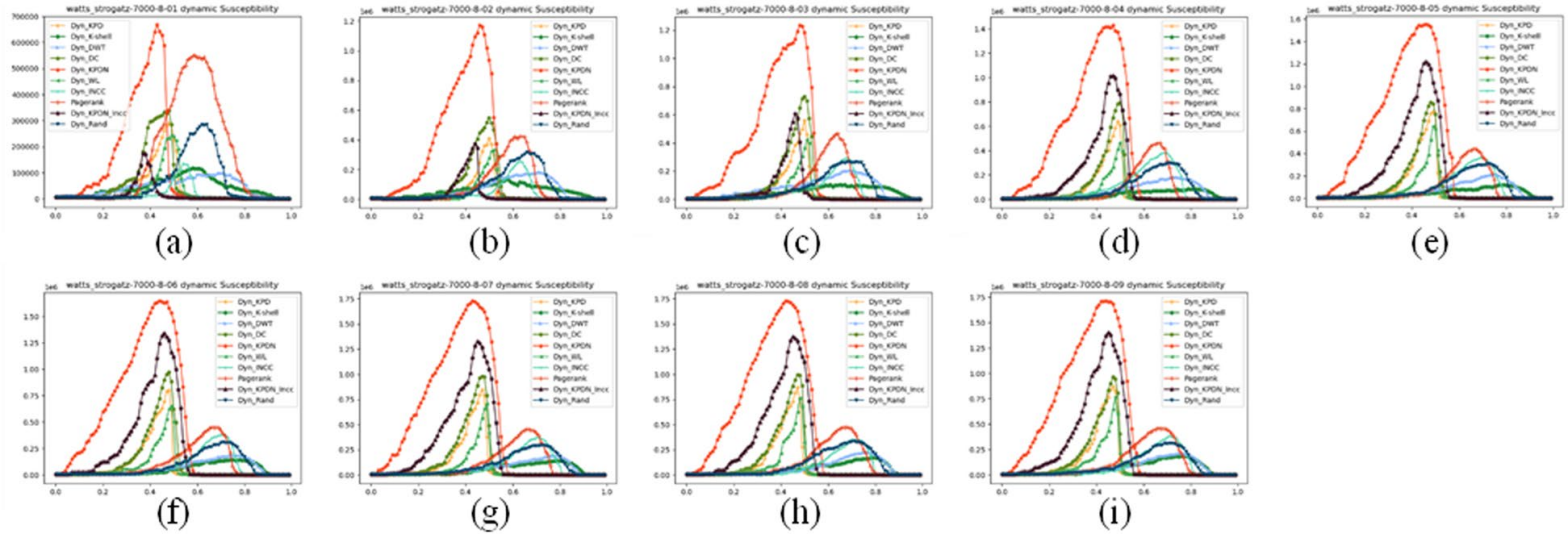
Subnetwork sensitivity of small-world networks under different attack strategies.

We also compared small-world effect networks. Small-world networks consist of three parts: the number of nodes, the average degree of each node, and the average random connectivity probability of the network. The number of nodes and the average degree of nodes affect the speed at which all methods quickly collapse, which is reflected in the image as the main change of the image tending towards the lower right or upper left. The results are shown in Figs. 7, 8 and 9. Figures a–i represent small-world networks with the same number of nodes, the same average node degree, and different average random connectivity probabilities of the network. The random connectivity probabilities are (a) 10%, (b) 20%, (c) 30%, (d) 40%, (e) 50%, (f) 60%, (g) 70%, (h) 80%, (i) 90%, respectively.

As shown in Fig. 8, as the random connectivity probability increases in small-world networks, small-world networks also gradually transition from regular networks to random networks. It can be seen from Fig. 8a–i that the KPDN–INCC method also has good performance in terms of the maximum remaining edges indicator.

Figure 9 shows the subnetwork sensitivity of small-world networks. From the peak point of each method’s graph, it can be clearly seen that the KPDN method and KPDN–INCC method have more significant results. The figure shows the high efficiency of the KPDN–INCC method and KPDN method in removing network nodes. (The highest point of subnetwork sensitivity represents the maximum destruction of the network by the attack method. The horizontal axis represents the proportion of nodes that need to be attacked or removed during destruction. Therefore, the smaller the corresponding horizontal axis value when reaching the highest point, the faster the network collapses and the better the effect.) It can be clearly seen from the figure that the KPDN–INCC method can quickly collapse the small-world network.

## Conclusion

This paper studies and proposes a dynamic important node identification and analysis method based on the KPDN method for complex networks under attack—KPDN–INCC. This method comprehensively considers the shortcomings of the existing KPDN method in failing to fully identify important nodes in a dynamic environment, and combines the improved network constraint coefficient (INCC) to make up for the deficiencies of the KPDN method in identifying dynamically important nodes in complex networks, especially for small-world networks with relatively small random connectivity probabilities. At the same time, we also analyze the possible limitations of the current methods. For example, more attention is currently paid to satisfying the maximum connected subgraph index, while other indicators may be sacrificed to some extent. It is hoped that the identification of important nodes in dynamic environments can be further improved and optimized in follow-up studies, such as comprehensively considering various network performance indicators to effectively identify important nodes during dynamic attacks, so as to support active defense and optimization of complex networks.

### Supplementary Information

Supplementary Information 1.

Supplementary Information 2.

Supplementary Information 3.

## Data Availability

All datasets mentioned in this paper can be obtained free of charge from https://networkrepository.com/networks.php.
