# Supplementary material for: Dynamic identification of important nodes in complex networks based on the KPDN–INCC method
Source: Sci Rep. 2024 Mar 9;14:5814. doi: 10.1038/s41598-024-56226-8 (PMC10924965; doi:10.1038/s41598-024-56226-8)
Supplement: Supplementary file 1 — Supplementary Information 1. [file 41598_2024_56226_MOESM1_ESM.docx]

Table 1. Calculation results of different methods for the demo case

| ID | Type | KPD | KPDN | Degree | K-shell | INCC | KPDN-INCC |
| --- | --- | --- | --- | --- | --- | --- | --- |
| 0 | A | 14.43 | 5.25 | 9.00 | 5.00 | 2.11 | 1394.41 |
| 1 | A | 14.54 | 4.27 | 9.00 | 5.00 | 2.39 | 626.54 |
| 2 | A | 12.30 | 2.40 | 7.00 | 5.00 | 1.72 | 557.89 |
| 3 | A | 13.29 | 3.85 | 8.00 | 5.00 | 1.97 | 487.28 |
| 4 | A | 10.07 | 0.85 | 5.00 | 5.00 | 1.59 | 294.18 |
| 5 | A | 12.20 | 2.82 | 7.00 | 5.00 | 1.55 | **11291.05** |
| 6 | A | 14.64 | 4.48 | 9.00 | 5.00 | 2.11 | 2390.37 |
| 7 | A | **16.65** | 7.83 | **11.00** | 5.00 | 2.46 | 2460.13 |
| 200 | B | 14.43 | 21.40 | 9.00 | 5.00 | 2.19 | 7242.48 |
| 201 | B | 14.77 | 4.18 | 9.00 | 5.00 | 2.46 | 1520.00 |
| 202 | B | 13.57 | 2.91 | 8.00 | 5.00 | 2.20 | 381.95 |
| 203 | B | 14.64 | 4.70 | 9.00 | 5.00 | **2.61** | 525.73 |
| 204 | B | 14.64 | 4.37 | 9.00 | 5.00 | 2.47 | 426.94 |
| 205 | B | 13.38 | 3.95 | 8.00 | 5.00 | 1.82 | 2702.12 |
| 206 | B | 8.20 | 0.42 | 4.00 | 4.00 | 1.55 | 39.35 |
| 207 | B | 14.39 | **22.11** | 9.00 | 5.00 | 1.81 | 7808.92 |
| 300 | C | 2.20 | 0.00 | 1.00 | 1.00 | 1.00 | 10.63 |
| 302 | C | 2.20 | 0.00 | 1.00 | 1.00 | 1.00 | 22.95 |
| 400 | D | 11.17 | 1.59 | 6.00 | 5.00 | 1.64 | 266.35 |
| 401 | D | 8.20 | 0.51 | 4.00 | 4.00 | 1.30 | 84.48 |
| 402 | D | 11.07 | 2.09 | 6.00 | 5.00 | 1.67 | 220.94 |
| 403 | D | 10.06 | 0.95 | 5.00 | 5.00 | 1.45 | 3746.57 |
| 404 | D | 12.22 | 3.62 | 7.00 | 5.00 | 1.67 | 607.47 |
| 301 | C | 0.00 | 0.00 | 0.00 | 0.00 | 0.00 | 0.00 |
| 303 | C | 0.00 | 0.00 | 0.00 | 0.00 | 0.00 | 0.00 |
| 304 | C | 0.00 | 0.00 | 0.00 | 0.00 | 0.00 | 0.00 |

Through the table data, it can be seen that the KPD method is based on certain improvements to the K-shell method, and the differentiation degree of nodes has been significantly improved. The most influential node for the KPDN method is node 207. The best ranked node for the Degree method is node 7. The K-shell method does not perform very well in this case, with a large number of nodes at the relatively high $Ks=5$ level, which is not conducive to identifying the importance of high-order nodes. The most influential node identified by the INCC method is node 203. And the most influential node proposed by our KPDN-INCC method in this chapter is node 5.

Here we focus on introducing the INCC method, the improved network constraint coefficient. The INCC method proposes node 203 as the optimal node. We can count out that node 203 forms 10 triangles in total with: $\Delta_{203,204,7}$,$\Delta_{203,204,200}$,$\Delta_{203,204,6}$,$\Delta_{203,204,202}$,$\Delta_{203,204,201}$,$\Delta_{203,7,200}$,$\Delta_{203,200,206}$,$\Delta_{203,200,201}$,$\Delta_{203,6,202}$,$\Delta_{203,6,201}$. Therefore, this method can better identify the number of edge triangles each node participates in the original network. Of course, Table 1 only shows the results of one time, and in subsequent processes, we re-evaluate the current network every step, collecting the top ranked value each time, and finally obtain our dynamic attack node order table through multiple dynamic attacks. The results of various methods are shown in Table 2. In order to have a more intuitive display of the final effects of dynamic attacks, we show the status of the remaining networks after attacking 13 nodes under various methods, as shown in Figure 3.

Table 2. Ranking results of important nodes by different dynamic methods for the demo network

| Node Ranking | KPD | KPDN | Degree | K-shell | INCC | KPDN-INCC |
| --- | --- | --- | --- | --- | --- | --- |
| 1 | 7 | 207 | 7 | 403 | 203 | 5 |
| 2 | 201 | 200 | 0 | 4 | 204 | 207 |
| 3 | 6 | 7 | 201 | 402 | 201 | 200 |
| 4 | 204 | 0 | 204 | 5 | 7 | 403 |
| 5 | 203 | 203 | 200 | 400 | 1 | 205 |
| 6 | 1 | 6 | 207 | 404 | 202 | 7 |
| 7 | 0 | 204 | 1 | 2 | 200 | 6 |
| 8 | 200 | 1 | 6 | 207 | 0 | 201 |
| 9 | 207 | 201 | 203 | 3 | 6 | 0 |
| 10 | 202 | 205 | 202 | 0 | 3 | 1 |
| 11 | 404 | 404 | 404 | 7 | 402 | 404 |
| 12 | 3 | 3 | 3 | 204 | 205 | 203 |
| 13 | 400 | 2 | 400 | 1 | 404 | 2 |
| 14 | 403 | 400 | 403 | 6 | 206 | 3 |
| 15 | 402 | 402 | 402 | 201 | 403 | 202 |
| 16 | 5 | 403 | 2 | 203 | 2 | 402 |
| 17 | 2 | 5 | 5 | 200 | 401 | 400 |
| 18 | 401 | 401 | 401 | 205 | 400 | 204 |
| 19 | 4 | 202 | 205 | 202 | 207 | 401 |
| 20 | 205 | 4 | 4 | 206 | 4 | 4 |
| 21 | 206 | 206 | 206 | 302 | 5 | 206 |
| 22 | 302 | 302 | 302 | 401 | 302 | 302 |
| 23 | 300 | 300 | 300 | 300 | 300 | 300 |

Since nodes 301, 303, and 304 did not participate in the overall connection of the network in the initial state, and the results of each method in the final dynamic node ranking remain 0, it shows that the importance of these three nodes is too low. Therefore, they are not included in the sorting process of each method in Table 2. At the same time, in order to have a more intuitive understanding of the method proposed in this paper and other existing methods, we show the remaining network after deleting 8 nodes and 13 nodes respectively based on the results of dynamically important nodes from the above six methods. As shown in Figure 3.
