# Supplementary material for: Dynamic identification of important nodes in complex networks based on the KPDN–INCC method
Source: Sci Rep. 2024 Mar 9;14:5814. doi: 10.1038/s41598-024-56226-8 (PMC10924965; doi:10.1038/s41598-024-56226-8)
Supplement: Supplementary file 3 — Supplementary Information 3. [file 41598_2024_56226_MOESM3_ESM.docx]

In this paper, we use several well-known metrics to study the performance of the proposed method. These metrics include:

(1)K-shell

The k-shell decomposition method proposed by Kitsak et al. which is a graph theory-based algorithm used to decompose an undirected graph into multiple k-shell subgraphs. A k-shell subgraph consists of nodes with a degree of at least k, meaning that the degree of any node within this subgraph is greater than or equal to k. The core idea of this algorithm is to iteratively prune the nodes with the smallest degree and update the degrees of their adjacent nodes until all nodes are decomposed into k-shell subgraphs.

(2) Degree Centrality (DC)

Degree centrality is a fundamental ranking algorithm used to identify the importance of nodes. The degree $k_{i}$ of node $i$ is defined as follows:

$$k_{i}=\sum_{j=1}^{N} a_{ij}$$

Here $a_{ij}$ represents the connectivity between node $i$ and node $j$. When there is a connection between nodes $i$ and $j$, $a_{ij}=1$. Conversely, when there is no connection between nodes $i$ and $j$, $a_{ij}=0$.

(3) Collective Influence (CI)

The Collective Influence of node $i$ is defined as follows:

$$CI_{i}=\left(k_{i}-1\right)\sum_{j\in set(i,l)} \left(k_{j}-1\right)$$

The $CI_{i}$ represents the collective influence of node $i$ relative to other nodes, and $k_{i}$ represents the degree value of node $i$.

(4) The WL algorithm

The WL algorithm is a network node importance method based on local features. It ranks nodes based on their own degree and the degrees of their neighboring nodes. The definition is as follows:

$$WL_{i}=\sum_{j\in N\left(i\right)} \left(k_{i}\times k_{j}\right)$$

The $WL_{i}$ represents the WL value of node $i$, and $k_{i}$ represents the degree value of node $i$. N(I) represents the set of neighboring nodes of node $i$.

(5) Harmonic Centrality (HC）

The harmonic centrality method (HC) [59] is defined as follows:

Here, $L\left(i,j\right)$ represents the shortest path length from node a to node b.

(6) INCC

The Improved network constraint coefficient (INCC) [59] is defined as follows:

$$INCC_{i}=\sum_{j} \left(\mu'_{ij}+\sum_{q\in\Gamma(i)\cap\Gamma(j)(q\neq i,j)} \mu'_{iq}\mu'_{qj}\right)^{2},\mu'_{ij}=\frac{Q_{j}}{LR_{i}}$$

Where QJ represents the sum of the first-order neighbors of node j, and LRI represents the sum of the second-order neighbors of node i.

(7) Pagerank

The PageRank method [60] is an algorithm proposed by Google's co-founders, Page and Brin, for ranking web pages. The specific formula is as follows:

$$PR\left(i\right)=\frac{\left(1-d\right)}{n}+d*\sum_{j\in N\left(i\right)} \frac{PR\left(j\right)}{C\left(j\right)}$$

Where $PR\left(i\right)$ and $PR\left(j\right)$ represent the PageRank values of nodes $i$ and $j$, $C\left(j\right)$ represents the out-degree of node $j$, $d$ is the damping factor typically set to 0.85, $n$ is the total number of web pages, and $N\left(i\right)$ represents the set of neighboring nodes of node $i$.

(8)DWT

The DWT algorithm is a decomposition method proposed by Ruan Yirun et al., which quantifies the strength of links based on local information of network topology. They also designed a simple and effective approach to assess node importance based on the node's degree and neighbor overlap.

$DWT_{i}=\sum_{j\in N\left(i\right)} \left(\frac{1}{k_{i}}+\frac{S_{ij}}{k_{i}}\right)^{2}$, $S_{ij}=\frac{\left|N\left(i\right)\cap N\left(j\right)\right|}{\sqrt{k_{i}\times k_{j}}}$

(9)random

Ranking the importance of network nodes through random scoring.

**References**

1. Kitsak M.; Gallos L. K.; Havlin S. et al. Identification of influential spreaders in complex networks. Nature Physics 2010, 6, 11.

2. Bonacich P. Factoring and weighting approaches to status scores and clique identification. The Journal of Mathematical Sociology, 1972, 1. doi:10.1080/0022250x. 1972. 9989806.

3. Morone F.; Min B.; Bo L.; Mari R.; Makse H.A. Collective Influence Algorithm to find influencers via optimal percolation in massively large social media. Scientific reports 2016, 6, 1. doi:10.1038/srep30062.

4. Wang J.W.; Rong L.L.; Guo T.Z. A new measure method of network node importance based on local characteristics, Journal of Dalian University of Technology 2010, 50, 822–826.

5. Massimo M.; Vito L. Harmony in the small-world. Physica A: Statistical Mechanics and its Applications 2000, 3. doi:10.1016/S0378-4371(00)00311-3.

6. D.Y.Zhang, Y.Wang, Z.X.Zhang. Identifying and quantifying potential super-spreaders in social networks.[J]. Scientific reports,2019,9(1).

7. Y.R.Ruan, J.Tang,Y.L.Hu, et al. Efficient Algorithm for the Identification of Node Significance in Complex Network[J]. IEEE Access,2020,8.

8. Qi Z ,Rongxia T ,Zhengan Y , et al.A parallel PageRank algorithm for undirected graph[J].Applied Mathematics and Computation,2023,459.
